# Combined Methylprednisolone Pulse Therapy plus Rituximab for Treating a Rare Juvenile Steroid-Resistant Nephrotic Syndrome with Cerebral Venous Sinus Thrombosis: A Case Report

**DOI:** 10.3390/jcdd9110383

**Published:** 2022-11-08

**Authors:** Hader I. Sakr, Burhan Edrees, Hussein Omar Taher, Tuleen Talal Miliany, Raneem Yasser Gazzaz, Asma Omar AlRuwaithi, Mohammed Fouad Alamer, Mostafa E. Metawee

**Affiliations:** 1 Department of Medical Physiology, Faculty of Medicine, Cairo University, Cairo 11511, Egypt; 2 Medicine Program, Batterjee Medical College, P.O. Box 6231, Jeddah 21442, Saudi Arabia; 3 Department of Pediatrics, Umm Al-Qura University, Makkah 24451, Saudi Arabia; 4 Department of Histology and Cytology, Faculty of Medicine, Al-Azhar University, Cairo 11511, Egypt

**Keywords:** nephrotic syndrome, steroid-resistant, cerebral venous sinus thrombosis, methylprednisolone, Rituximab

## Abstract

**Background**: Cerebral venous sinus thrombosis (CVST) secondary to nephrotic syndrome (NS) is rarely reported. Additionally, treating steroid-sensitive nephrotic syndrome (SSNS) that changes to steroid resistance (SRNS) is difficult, with many relapses and side effects. **Case presentation**: A 32-month-old SSNS male child turned into SRNS and developed cerebral venous sinus thrombosis (CVST), a rare complication of NS. As a result of the administration of combined pulse methylprednisolone and IV Rituximab (RTX) therapy, the patient showed marked improvement, the results of urine analysis were remarkably improved, and the child started to respond to treatment. **Conclusions**: Successful treatment of a rare case of juvenile SSNS behaving as SRNS with the development of CVST could be established using combined steroid pulse therapy, Enoxaparin, and the B lymphocytes monoclonal antibodies RTX.

## 1. Introduction

Nephrotic syndrome (NS) is considered the most common clinical manifestation of a series of primary and secondary glomerulonephritides during childhood, with a prevalence of 12–16 per 100,000 children under 16 [1]. The reported incidence in children is about 4.7 per 100,000 per year [2]. About 80% of children showing complete remission within four weeks after corticosteroid treatment is considered a steroid-sensitive nephrotic syndrome (SSNS). Although more than 85% of children with NS respond to corticosteroids, 60%–70% of the patients with original SSNS have more than one relapse [3], and the remaining 10–15% remain nonresponders or later change to steroid-resistant (SR) [4,5]. Steroid resistance is considered when there is a lack of remission despite four weeks of 2 mg/kg daily prednisone therapy [6]. SRNS usually shows a poor prognosis, as 36–50% of patients can develop end-stage kidney disease during the first ten years [3].

NS is a clinical condition characterized by hyperlipidemia, proteinuria, hypoalbuminemia and oedema. It is usually accompanied by hypercoagulability leading to thrombosis, most frequently in the lower extremities’ deep veins and renal veins [7], but the development of cerebral venous sinus thrombosis (CVST) is considered a rare complication in NS.

Pulse IV methylprednisolone was first recommended for the treatment of SRNS by Mendoza and Tune in 1990 [8]. However, prolonged steroid therapy can result in significant side effects such as hypertension, diabetes mellitus (DM), growth impairment, obesity, cushingoid facies, abdominal striae, osteoporosis, cataracts, immune suppression, psychosis, and hirsutism [3]. **Calcineurin inhibitors** (CNIs) were used to treat SRNS [9]. The rates of partial or complete remission with CNIs in SRNS were 30–80% in observational studies [10] and randomized controlled trials. Combined steroids and CNIs for treating SRNS proved potency in systematic reviews [11]. Ehrich et al. demonstrated that prolonged combined therapy with high-dose methylprednisolone and **cyclosporine** kept the remission rate for patients with SRNS at 84% [12]. Cyclosporine therapy has remarkable side effects such as nephrotoxicity, hypertension, gum hypertrophy, and hypertrichosis [3]. **Tacrolimus** is considered an alternative to cyclosporine with less gum hypertrophy and hypertrichosis. However, other adverse effects were reported as tremors, hypertension, and DM [13]. **Mycophenolate mofetil** (MMF) may seldom be used as a substitute for treating SRNS in patients with serious adverse effects on CNIs. MMF has several side effects, such as metabolic acidosis, abdominal pain, diarrhea, infection, and hyperlipidemia [14].

Rituximab (RTX) is a chimeric anti-CD20 monoclonal antibody used as a well-tolerated steroid-saving alternative for patients with SSNS for about two decades. Its immune-regulating action targets the CD20 antigen on B-lymphocytes’ surface, causing cell depletion [15]. Nevertheless, another immune-independent mechanism of action was suggested. RTX was reported to affect podocytes’ functions by stabilizing sphingomyelin phosphodiesterase acid-like 3b (SMPDL-3b), preventing the remodeling of podocytes’ actin [16]. In general, RTX was used successfully in children with SRNS [6,17,18,19]. A recent study reported complete remission (100%) in SRNS children receiving RTX. The timing of RTX initiation in SRNS patients is very crucial [20]. Two standard doses of RTX failed to achieve remission in refractory SRNS patients within three months after administration in a previous randomized-control trial [21]. In a different study, three months of RTX administration with repeated doses successfully induced remission in most patients. Repeated administration over an extended duration can be successful in case of unresponsiveness in SRNS [20]. Evidence supports that persistent urinary losses in SRNS may decrease serum RTX levels more rapidly [22,23]. In that regard, more RTX doses are required in SRNS patients with uncontrolled proteinuria [20,24].

## 2. Case Presentation

A 32-month-old male child previously diagnosed with steroid-sensitive nephrotic syndrome (SSNS) on alternate-day methylprednisolone 1 mg/kg/day therapy was presented to the pediatric outpatient clinic of the Saudi-German Hospital, Jeddah, KSA, on 14 November 2021 with persistent relapse secondary to 4 days of mild upper respiratory tract infection (RTI). His parents, a Pakistani father and a Tunisian mother, had no family history of similar diseases. The child had a history of frequent relapses during the previous 12 months after responding to steroid treatment which cannot be stopped as he relapsed during tapering every other day. This rendered the child steroid sensitive but dependent. On examination, the child was crying and irritable with puffy eyes. Blood pressure (BP) was 99/72, heart rate (HR) was 90/min, respiratory rate (RR) was 20/min, body weight was 18 kg at 95 centiles for age, and height was 100 cm at about 95 centiles for age. Lab investigations were ordered, including CBC, serum albumin and creatinine, urea (BUN), and urine analysis. Low serum albumin and 3+ (100–300 mg) urine protein results were suggestive of relapsed nephrotic syndrome (NS) with acute nasopharyngitis (common cold). Symptomatic Medications were prescribed, and the patient was switched to daily oral methylprednisolone 2 mg/kg, instructing the family to follow up urine for proteins. On 16 November 2021, follow-up urine analysis showed protein +3, and serum albumin was 1.77 g/dL (*n* = 3.4–5). The patient continued on daily oral steroids with calcium and Vitamin D supplement for three months.

**Figure 1:**
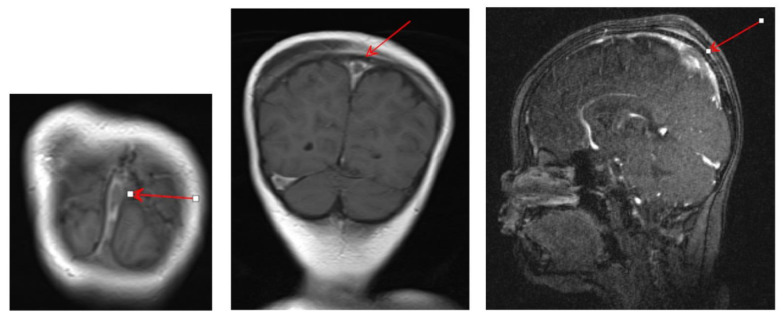
MRI and Contrast-enhanced MRV revealed filling defects (red arrows) within the superior sagittal sinus, reflecting thrombosis.

On 20 December 2022, the patient presented to the ER with severe headache, vomiting, diarrhea, and blurring of vision for one day. On examination, the patient appeared drowsy, with a high BP for his age (112/74). RR was 24/min, HR was 104/min, height was 103 cm, and weight was 22 kg (the weight gaining was attributed to the generalized oedema). Investigations including ESR, serum albumin, blood gases, pH, random blood glucose (RBS), urine analysis, CRP and d-dimer were ordered, and a contrast-enhanced brain MRI and MRV with gadolinium were performed. d-dimer was elevated and measured at 12.14 mg/L FEU (N > 0.50 mg/L FEU). MRI and MRV revealed absent enhancement in the right transverse and sigmoid sinuses and the posterior aspect of the superior sagittal sinus, with filling defects seen in the lumen of the distal aspect of the left transverse sinus suggestive of dural sinuses thrombosis (Figure 1). The child was admitted to the pediatric intensive care unit for management in the form of Enoxaparin (a low molecular weight form of heparin) 1 mg/kg every 12 h, switching the oral dose to full-dose IV steroid and antihypertensive medication in the form of Renitec (ACE-I) and Trandate (labetalol). By this time, the biopsy was risky due to the added anticoagulant therapy. Routine evaluation for genetic mutations is not recommended due to negative family history and the variable availability of genetic testing, significant cost, low to absent prevalence observed in some populations, and the lack of systematic studies of treatment response and prognosis relative to specific genetic polymorphisms.

**Figure 2:**
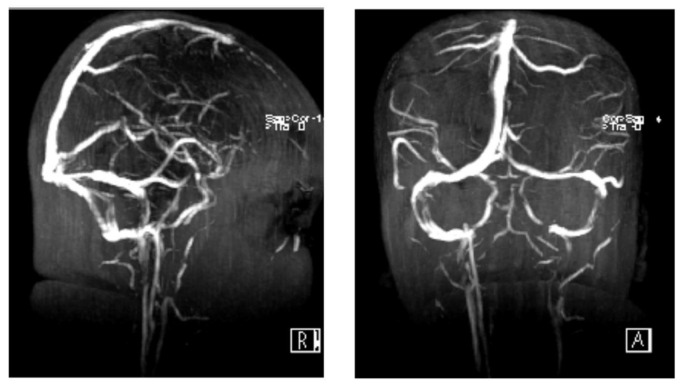
Contrast-enhanced MRV reveals a normal appearance of dural venous sinuses with no evidence of dural venous thrombosis.

On 27 December 2022, D-dimer was ameliorated and measured at 0.45 mg/L FEU. Repeated MRV showed interval improvement compared to the previous exam in the form of decreased size of the previously seen extensive venous sinus thrombosis with slight partial recanalization. Urine analysis daily repetition was advised (Figure 2).

On 28 February 2022, the patient showed continuous non-responsivity to steroids after completing eight weeks’ steroid full dose, rendering him steroid-resistant (SRNS). As per the nephrologist, IV Rituximab (RTX) 375 mg/m^2^ once for four monthly doses was started, and as per ID pre-dose, Quantiferon evaluation for TB was negative. Three-day pulse therapy methylprednisolone 30 mg/kg/dose was also given simultaneously. The patient improved with decreasing proteinuria, and by the third day, the urine dipstick was negative, as confirmed by the lab. As per the pediatric hematologist, Enoxaparin therapy dose adjustment started after checking the anti-factor Xa activity. By this combination, the child responded and achieved remission, which is considered unique, and this patient condition is known as “SSNS behaves as SRNS” and was transferred back to SSNS [17].

On 2 March 2022, the patient’s urine analysis showed negative protein, bacteria, and pus, emphasizing improvement after pulse prednisolone therapy and RTX. On 6 March 2022, the patient was discharged on Renitec 10 mg orally once daily for one month, Trandate oral syrup 10 mg 3 times a day for one month, oral prednisolone for one month with tapering instruction, and Enoxaparin 1 mg/kg twice a day. On 15 March 2022, the patient’s follow-up confirmed responsivity with negative urine protein. During the subsequent follow-up on 6 April 2022, the doctor planned to give extra three doses of RTX, with the first extra dose received on 7 April 2022. Follow-up on 19 April 2022; the ordered lab investigations in the form of CBC, renal panel, LFT, and CRP were all normal. Steroid tapering was continued by reducing the dose by 1 mL/week. The patient reached a dose of 5 mL every other day on 28 June 2022, after which the dose was further reduced by 1 mL every two weeks. On 26 July 2022, the dose became 3 mL every other day without relapses.

## 3. Discussion & Conclusions

Steroid-resistant nephrotic syndrome (SRNS) is considered a defy facing pediatric nephrologists. SRNS is a disease of heterogeneous nature, including monogenic and immune-based etiologies. Being a relatively rare condition, the treatment planes of SRNS vary among nephrologists and centers of expertise [9].

Glucocorticoids (GCs) can directly promote podocyte repair and protect the podocytes from injury via stabilizing actin filament and preventing apoptosis [25]. GCs upregulate nephrin expression, a vital constituent of the slit diaphragm that is considered the primary site regulating glomerular permeability [26]. The GCs affect apoptosis and nephrin in a dose-dependent manner, which may explain the vast diversity of clinical responses to GCs [27]

NS is commonly complicated with thrombosis, frequently in the form of deep and renal venous thrombosis and pulmonary embolism manifested by fever, waist pain, limb swelling, and visible hematuria. Thrombosis in NS is caused mainly by hypercoagulability, infections (pneumonia, urinary tract infection and peritonitis) and diuretic abuse [28,29]. CVST is a much less frequent complication in pediatric NS, with a rate of 0.67 per 100,000 [30]. CVST has multiple non-specific clinical presentations in the form of decreased consciousness, dizziness, headaches, vomiting, papilledema, and twitching, rendering it misdiagnosed as children usually fail to express discomfort [31]. CVST risk factors can be either infectious (central nervous, oral, facial, and sinus infection) [32] or non-infectious (cerebral tumors, head traumas, internal jugular veinous malformations, dural arteriovenous fistulas, systemic vascular diseases, among others) [33,34]. Prethrombotic factors include deficiency of anti-thrombin, protein C, and protein S, high levels of factor VIII and factor V Leiden [35]. The patient’s condition can advance within a few hours or days, and it can be fatal in critical cases. Imaging is considered the diagnostic tool of choice. Generally, head MRI and MRV examinations can diagnose CVST. MRV is also used for CVST follow-up [36]. Treatment is essentially through anticoagulant therapy such as urokinase, low molecular weight heparin, and warfarin, either individually or in conjunction [37].

The reason for adding intravenous pulse methylprednisolone administration despite the initially developed steroid resistance is based on the reported remission results from different NS observational studies. Remission induction in SRNS was reported by using methylprednisolone either alone or methylprednisolone pulses with concomitant immunosuppressants [38]. The prolonged methylprednisolone pulses combined with oral steroids every other day may be effective, even in patients having steroid- and multidrug-resistant NS [9]. A long-standing NS leads to irreversible histologic changes in the form of fibrosis and glomerulosclerosis accompanied by inadequate response to RTX treatment [17,20].

RTX was initially introduced as a lymphoma and autoimmune diseases treatment [39]. Many reports introduced it as a treatment strategy for SDNS children. RTX appears to have effectiveness in reducing relapses and decreasing the required steroid dose in children with refractory NS [40]. Moreover. many authors strongly suggested that RTX could be an effective treatment in SRNS and may induce disease remission [18].

The rationale for using RTX is mainly its B lymphocytes’ anti-CD20 antigen effect. CD20 is expressed on the cell membrane of B cells during the B lymphocyte central phase of development. The depletion of CD20+ effectors and memory B lymphocytes blocks the activation chain that produces allo- and auto-antibody. At the same time, early B cell precursors are exempted from this effect, allowing immature cells to regenerate new B cells with antibodies lacking pathogenic autoreactivity. Long-term defensive antibody memory is possible due to mature plasma cells sparing as well [41]. Another B-lymphocyte-independent mechanism is through stabilizing podocytes’ cytoskeleton directly, which improves proteinuria [42].

To summarize, GCs are the treatment of choice in NS through their genomic and non-genomic actions. SRNS resembles a defy for pediatric nephrologists. CVST is a rare and less frequent complication in pediatric NS that could be diagnosed through head MRI and MRV examination and is usually treated using anticoagulant therapy. Even though RTX treatment in SRNS does not have the same efficacy as in SSNS, favorable renal results resulted from early and repeated administration of RTX with higher cumulative doses and methylprednisolone pulses with high-dose prednisolone.

## Data Availability

The dataset generated in the current study are available from the corresponding author upon request.
